# IMPACT OF OLDER ADULT PATIENTS AND CAREGIVER ATTITUDES AND PERCEPTIONS AND LOCAL CULTURE ON IN-HOSPITAL MOBILITY

**DOI:** 10.1093/geroni/igae098.1790

**Published:** 2024-12-31

**Authors:** Barbara King, Anna Zisberg, Cynthia Brown

**Affiliations:** Chair; Co-Chair; Discussant

## Abstract

Older adults commonly lose functional ability during a hospital stay, often cause by limited ambulation. This phenomenon has been identified globally as a hospital associated disability (HAD). Multiple barriers related to how care is delivered in hospitals have been identified that prevent hospital staff from getting patients up to walk. Only recently have studies explored other factors that may prevent or influence whether older patients ambulate, including older adults’ experiences and perceptions of in-hospital ambulation, the impact of patient and or family caregiver attitudes on in-hospital mobility, and influence of local unit culture on patient mobility. This symposium will present results from hospitalized older adult mobility studies conducted in 3 countries. Paper 1 will describe older adult patient perceived Value for Walking during hospitalization; paper 2 will discuss the impact of patient and family attitudes on patient mobility; paper 3 will present how diversity and cultural difference of family caregivers impacts patient mobility; and paper 4 will describe how local unit culture mediates mobility of older adult patients. HAD in older adults is a global health epidemic. Designing effective person-centered mobility interventions need to be multifactorial and include patient and family/ caregiver perspective and unit culture in order to improve older adult patient ambulation and decrease the incidence of HAD. Additional research is needed to understand the impact of patient and family caregiver factors and local unit culture on whether older adult patients are engaged in walking during their hospital stay.

